# Antimicrobial Peptide Sublancin Skin Sensitization and Irritation Assessment in Guinea Pigs and Rabbits

**DOI:** 10.3390/toxics14010069

**Published:** 2026-01-12

**Authors:** Yong Guo, Lin Zhang, Gantong Guo, Tao He, Yangke Liu, Yujiao Lai

**Affiliations:** 1 School of Chemistry and Environmental Engineering, Wuhan Institute of Technology, Wuhan 420200, China; 11809010003@stu.wit.edu.cn; 2 Sinagri YingTai Bio-peptide Co., Ltd., Linzhou 456550, China; znytggt@126.com (G.G.); znytht@126.com (T.H.); znytlyk@126.com (Y.L.); 3 Key Laboratory of Feed Antibiotics Replacement Technology, Ministry of Agriculture and Rural Affairs, Linzhou 456550, China; 4 Hubei Key Laboratory of Animal Nutrition and Feed Science, Engineering Research Center of Feed Protein Resources on Agricultural By-Products, Ministry of Education, Wuhan Polytechnic University, Wuhan 430023, China; 17809057814@163.com

**Keywords:** sublancin, antimicrobial peptide, skin sensitization, skin irritation, safety evaluation, Guinea Pig Maximization Test (GPMT)

## Abstract

This study evaluated the skin sensitization of the antimicrobial peptide sublancin to support its safety assessment for topical application. Sensitization was assessed using the guinea pig maximization test (GPMT), in which animals received sublancin (2 mg/kg), vehicle (negative control), or 2,4-dinitrochlorobenzene (positive control) during induction and challenge phases. Skin reactions (erythema and edema) were recorded after challenge. Irritation was evaluated in rabbits following single and repeated applications of sublancin to intact and abraded skin, with observations made at multiple time points. In the GPMT, no erythema or edema was observed in the sublancin-treated group or negative control group at 24, 48, and 72 h post-challenge, corresponding to a sensitization rate of 0%. All animals in the positive control group exhibited moderate to severe erythema and edema (sensitization rate 100%). In both single- and repeated-dose rabbit irritation tests, sublancin induced no erythema or edema on intact or abraded skin at any observation point, resulting in a total irritation score of 0. Furthermore, no significant differences in the daily weight gain were observed between any experimental group and the negative group (*p* > 0.05). In conclusion, under the conditions of this study, sublancin showed no skin sensitization potential in guinea pigs and no irritant effects in rabbits, supporting its local tolerance for topical veterinary use.

## 1. Introduction

The intensification of modern animal husbandry has led to the widespread use of antibiotics, leading to serious challenges including bacterial resistance and drug residues that threaten animal health, food safety and public health [1,2,3]. In response, China has implemented policies promoting the “reduction and replacement” of antibiotics, explicitly encouraging the development of safe and efficient novel alternatives [4]. Antimicrobial peptides (AMPs) are considered one of the most promising candidates due to their broad-spectrum antimicrobial activity, unique mechanisms of action, and low propensity for inducing resistance [5,6].

Sublancin, an AMP produced by Bacillus subtilis 168 [7], exhibits potent inhibitory activity against various Gram-positive bacteria [8] and demonstrates additional biological functions such as immunomodulation and gut barrier protection [9,10,11]. Importantly, a recently completed systematic toxicology study established a foundational safety profile for systemic exposure: in subchronic and chronic toxicity studies in SD rats, doses as high as 50,000 mg/kg feed induced no drug-related systemic toxicity, confirming a No-Observed-Effect Level (NOEL) of 50,000 mg/kg [12]. This result has laid a solid foundation for the safety evaluation of systemic exposure.

However, systemic safety does not guarantee local tolerance for topical applications. For veterinary products intended for skin use (e.g., teat dips, wound sprays), assessing local effects is a critical and mandatory step in safety evaluation [13]. Skin sensitization tests evaluate a substance’s potential to induce allergic contact dermatitis after repeated exposure [14], while irritation tests focus on reversible local inflammatory damage (such as erythema and edema) following a single or short-term application [13]. These assessments form the core safety basis for topical products and are explicit requirements for veterinary drug registration.

Therefore, a standardized evaluation of local tolerance is essential early in the development of new veterinary topical formulations. This study, following standardized toxicological guidelines, systematically evaluated the skin sensitization and irritation potential of sublancin using the Guinea Pig Maximization Test (GPMT) and rabbit skin irritation tests, respectively. The work addresses a key gap in its dermal safety assessment. Together with existing systemic toxicity data, it forms a complete safety evidence chain from local to systemic exposure. Furthermore, it provides a standardized practical framework for the preclinical safety evaluation of novel AMP-based preparations and supports their subsequent industrial development.

## 2. Materials and Methods

### 2.1. Test Substances and Characterization

The test substance, sublancin, is a bacteria-derived glycopeptide (lantibiotic antimicrobial peptide) produced and isolated by Bacillus subtilis 168. It is composed of 37 amino acid residues. Its molecular weight is approximately 3879.8 Da. The three-dimensional solution structure of this peptide was analyzed by nuclear magnetic resonance (NMR) spectroscopy and exhibited a spatial conformation characterized by intramolecular disulfide bonds and specific O-glycosylation (structural diagram is shown in Figure S1 of Supplementary Materials) [15]. The sublancin used in this study was a purified preparation, with a purity of 93.0% on a dry basis.

### 2.2. Chemicals and Reagents

Sublancin was supplied by Linzhou Sinagri Yingtai Bio-peptide Co., Ltd. (Linzhou, China). The positive control substance, 2,4-dinitrochlorobenzene (DNCB, purity > 99%), was purchased from Sigma-Aldrich (St. Louis, MO, USA).

### 2.3. Experimental Animals

Hartley strain albino guinea pigs (weight range: 260–320 g, half male and half female) were purchased from Beijing Haidian Xingwang Animal Farm. A total of 18 New Zealand White rabbits (weight range: 2.5–3.0 kg, half male and half female) were obtained from Beijing Haidian Xinglong Experimental Animal Farm. The specific housing conditions for each species were as follows. Guinea pigs were housed in plastic cages (80 cm × 50 cm × 20 cm) with five animals per cage in a standard conventional animal room. The ambient conditions were maintained at a temperature of 22 ± 4 °C, relative humidity of 55 ± 10%, with an artificial light/dark cycle, and noise levels below 60 dB. Bedding was changed twice a week. Rabbits were housed individually in stainless steel cages (50 cm × 30 cm × 30 cm) in a standard conventional animal room. The ambient conditions were maintained at a temperature of 18–22 °C, relative humidity of 40–60%, with a ventilation rate of ≥14 air changes per hour and adequate lighting.

All animals had ad libitum access to a standard diet (purchased from Beijing Ke’ao Xieli Feed Co., Ltd., Beijing, China) and purified water. All animal experiments were performed at the animal facility of the College of Veterinary Medicine, China Agricultural University. The experimental protocol was reviewed and approved by the Institutional Animal Care and Use Committee of Wuhan Polytechnic University (Approval No. WPU202211006), which authorized all procedures conducted at the collaborating site.

### 2.4. Experimental Procedures

#### 2.4.1. Dose Preparation and Rationale

The test substance (sublancin) was formulated as an aqueous suspension for topical application. The dose selected for both the skin irritation and sensitization assays was 2 mg/kg.

Rationale for Dose Selection: This dose was determined to simulate a clinically relevant, worst-case single dermal exposure from the intended veterinary use (e.g., as a topical spray). The calculation was based on a two-step rationale: (1) estimation of the maximum probable dermal load of the active ingredient during a full application, derived from a proposed product concentration (0.1% *w*/*v*) and typical application volume; (2) translation of this total load (~5–6 mg) into a standardized dose for the rabbit model, normalized by the average animal weight (~2.7 kg). Testing at this exposure level allows for a conservative assessment of local tolerance and provides a direct basis for establishing a safety margin relative to the effective dose.

#### 2.4.2. Skin Sensitization Test Protocol

Forty healthy guinea pigs were randomly divided into three groups (equal numbers of males and females per group): a test substance group (sublancin, *n* = 20), a negative control group (*n* = 10), and a positive control group (DNCB, *n* = 10). Twenty-four hours before the experiment, a 4 cm × 6 cm area on the dorsal neck region of each animal was closely clipped, taking care to keep the skin intact.

Induction Phase: Conducted on days 0, 7, and 14. For the test substance group, sublancin (2 mg/kg) was applied topically to the left clipped area. The positive control group received 0.2 mL of a 0.6% (*w*/*v*) DNCB solution, and the negative control group remained untreated. The applied materials were covered with gauze and non-irritating occlusive film, secured with adhesive tape for 6 h, after which the patches were removed. Skin reactions were observed and recorded at 1 h and 24 h after each exposure.

Challenge Phase: Conducted on day 28. A challenge dose was applied to the contralateral (right) clipped region. The test substance and the negative control groups received topical sublancin, while the positive control group received 0.2 mL of a 0.3% (*w*/*v*) DNCB solution, using the same application and occlusion procedure as in the induction phase. After 6 h, patches were removed. Erythema and edema were observed and recorded at 24, 48, and 72 h post-removal according to the criteria in Table 1. The sensitization rate was calculated based on Table 2.

#### 2.4.3. Skin Irritation Test Protocol

Twelve healthy rabbits were randomly assigned to four groups (*n* = 3 per group): single-application intact skin, single-application abraded skin, repeated-application intact skin, and repeated-application abraded skin. Twenty-four hours before dosing, fur on both sides of the dorsal spine (approximately 3 cm × 3 cm per side) was closely clipped. For animals in the abraded-skin groups, the stratum corneum was lightly breached with a sterile needle immediately before substance application to produce a non-occlusive abrasion with minimal bleeding.

Single-Application Irritation Test: A volume of 0.5 mL of sublancin suspension, equivalent to 2.0 mg/kg body weight, was applied to the designated test areas. An equal volume of vehicle was applied to the contralateral control areas. Sites were covered with two layers of gauze and a non-irritating plastic film, then secured. After 4 h, patches were removed and residual substance was gently cleansed with lukewarm water. Skin reactions at test and control sites were observed and scored at 1, 24, 48, and 72 h after patch removal.

Repeated-Application Irritation Test: The test substance was applied once daily to the same site for seven consecutive days. Reactions were observed and scored at 1 h after each daily removal. Following the final (7th) application, additional observations and scoring were performed at 1, 24, 48, and 72 h post-removal. Skin irritations were evaluated using the scoring criteria in Table 3, and the overall irritation intensity was classified according to the grading system in Table 4.

#### 2.4.4. General Clinical Observations

The overall health status of all guinea pigs and rabbits was monitored daily by trained personnel. Detailed records were maintained for appearance, behavior, respiration, appetite, fur condition, and any abnormal secretions. Fecal consistency and color were noted. All animals were closely observed for toxic signs. In the event of severe illness or death, a post-mortem examination was conducted promptly. Skin conditions at application sites were meticulously observed and recorded at scheduled times before and after each administration.

#### 2.4.5. Body Weight Measurement and Skin Reaction Assessment

Body Weight Measurement: For guinea pigs enrolled in the 28-day skin sensitization study, body weight was recorded as a general health indicator at the beginning of the study (Day 0) and on a weekly basis thereafter using a calibrated electronic balance. Weight gain was calculated and analyzed statistically.

Skin Reaction and Clinical Assessment: All scoring of dermal reactions (erythema and edema) according to the specified criteria (Table 1 and Table 3), along with general clinical observations, was performed independently by two trained technicians. The assessors were blinded to the treatment groups and were experienced in conducting Guinea Pig Maximization Tests (GPMT) and acute dermal irritation studies in accordance with OECD guidelines. Any scoring discrepancies were resolved through consensus.

### 2.5. Statistical Analysis

Body weight data are presented as mean ± standard deviation (Mean ± SD). Differences between groups were analyzed using one-way analysis of variance (ANOVA). Statistical significance was set at *p* < 0.05. Data for skin irritation and sensitization endpoints were evaluated using descriptive statistics only and are presented as the incidence of response. All statistical analyses were performed using SPSS version 26.0 (IBM Corporation, Chicago, IL, USA). Graphical representations were generated using GraphPad Prism version 10.0 (GraphPad Software, San Diego, CA, USA).

## 3. Results

### 3.1. General Clinical Symptoms

Throughout the study, no remarkable changes in the general clinical sign, including behavior, mental state, appetite, feces, fur, respiration, and body weight, were observed in any treatment group of guinea pigs and rabbits compared to the negative control. No abnormal secretions from the nose, eyes, or oral cavity were noted. Critically, the skin at all application sites remained free of erythema, edema, or other signs of irritation for the duration of the administration and observation periods.

### 3.2. Body Weight

**Figure 1:**
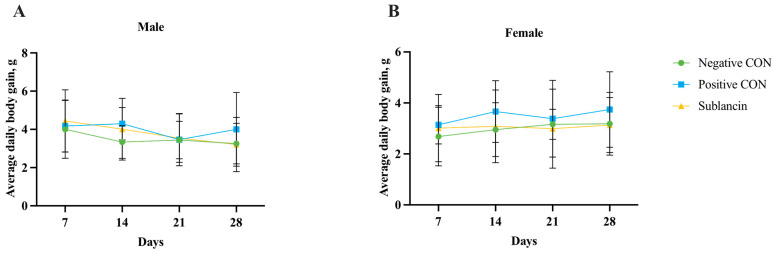
Body weight changes in guinea pigs over the experimental period. (**A**) Male. (**B**) Female.

Body weight, a key indicator of general health, was monitored throughout the study (Figure 1 and Table S1). Statistical analysis showed no significant difference in body weight gain between the sublancin-treated group (*n* = 20) and the negative control group (*n* = 10; *p* > 0.05). Similarly, body weight changes in the positive control group (*n* = 10) did not differ significantly from those in the negative control group (*n* = 10; *p* > 0.05), indicating that neither the test nor the positive control substance markedly affected overall animal growth under the experimental conditions.

### 3.3. Skin Sensitization Test

#### 3.3.1. Induction Phase

Following each of the three sensitizing exposures, no skin reactions (erythema or edema) or other abnormalities were observed in the sublancin-treated (2 mg/kg) or negative control groups at the 1 h and 72 h observation points. Animals in these groups maintained normal dietary intake, excretion, and behavior throughout this phase. In sharp contrast, the positive control group (DNCB) exhibited typical sensitization signs after each exposure, including agitation, pain, scratching, and the development of moderate erythema with mild edema.

#### 3.3.2. Challenge Phase

**Figure 2:**
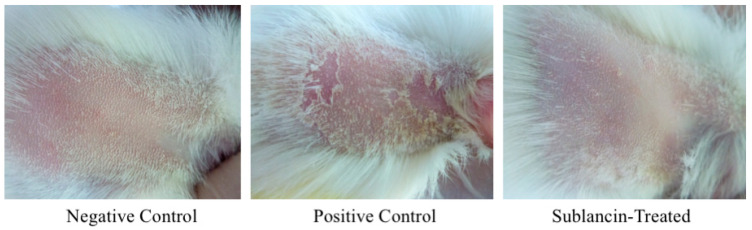
Comparison of skin reactions between the test group and control groups in the guinea pig skin sensitization study.

Skin reactions at 24, 48, and 72 h post-challenge are presented in Figure 2, with detailed scores and incidence rates provided in Table 5. No erythema or edema was observed at any time point in the sublancin-treated group (*n* = 20) or the negative control group (*n* = 10), resulting in a sensitization incidence of 0% for both. Conversely, all animals in the positive control group (*n* = 10) exhibited moderate to severe erythema and edema, corresponding to a 100% allergic reaction incidence. Animals in this group also displayed renewed signs of agitation, pain and itching during the challenge phase.

### 3.4. Skin Irritation Tests

#### 3.4.1. Single-Dose Application

**Figure 3:**
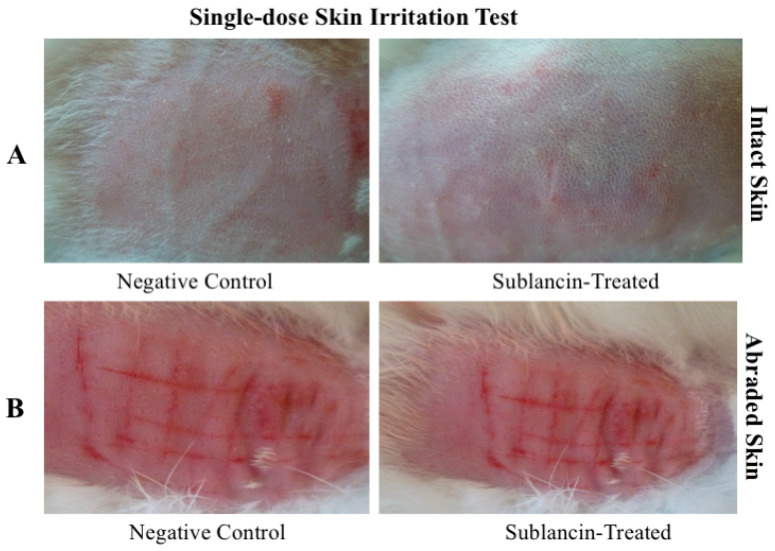
Representative photographs of rabbit skin from the single-dose irritation test. (**A**) intact and (**B**) abraded skin.

In the single-dose test on intact rabbit skin, sublancin induced no irritant response. As detailed in Table 6 and visualized in Figure 3A, no erythema or edema was observed at the application sites at any observation time (1, 24, 48 and 72 h). The total irritation score remained 0 for all animals, indicating no difference from control sites or pre-application skin. This confirms that sublancin is non-irritating to intact rabbit skin.

Table test on abraded skin yielded identical results. No signs of irritation were observed at any abraded application site during the entire observation period. The total irritation score remained 0 at all time points for all individuals (Table 7, Figure 3B), confirming that sublancin is also non-irritating to abraded rabbit skin.

#### 3.4.2. Repeated-Dose Application

**Figure 4:**
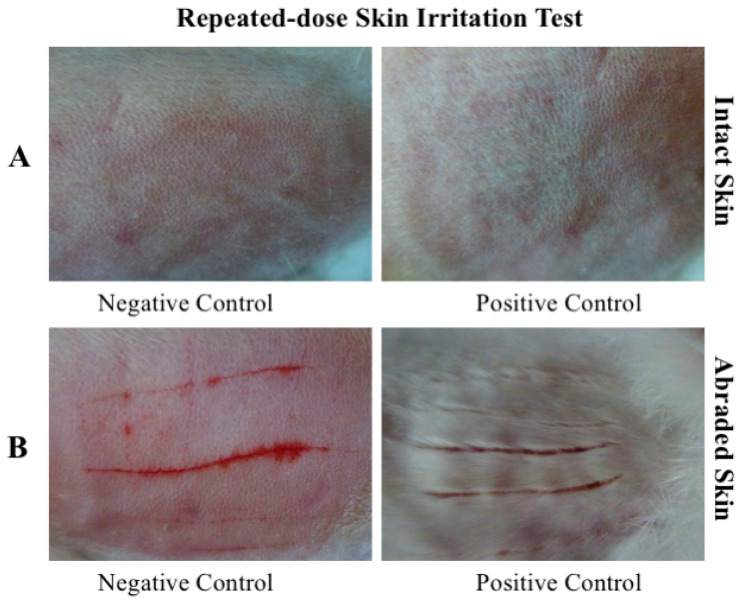
Representative photographs of rabbit skin from the repeated-dose irritation test. (**A**) Intact and (**B**) abraded skin.

Repeated application of sublancin over one week did not induce irritation on intact rabbit skin. No erythema or edema was observed following any application. Scores recorded after the final application confirmed a total irritation score of 0 for all animals at all time points (Table 8, Figure 4A).

Similarly, repeated application to abraded skin caused no irritation. As shown in Table 9 and Figure 4B, no erythema or edema was observed on test or control skin before or after any administration, or after the final dose. The total irritation score was consistently 0, confirming that repeated application of sublancin is non-irritating even to compromised skin.

## 4. Discussion

This study, in accordance with the Technical Guidelines for Veterinary Drug Research issued by the Ministry of Agriculture and Rural Affairs of China and the OECD Test Guidelines (TG 404 and 406) [16,17], systematically evaluated the topical safety profile of the antimicrobial peptide sublancin through standardized GPMT and acute dermal skin irritation test in rabbits. The core experimental outcomes consistently demonstrated an absence of skin irritation and sensitization potential under the tested conditions. The following discussion synthesizes these findings, moving beyond a mere restatement of results to explore their mechanistic underpinnings, regulatory significance, and translational implications for developing sublancin as a topical veterinary agent.

The reliability of the concluded safety profile is fundamentally underpinned by the appropriate responsiveness of the employed toxicological models. In the rabbit skin irritation test, sublancin elicited no erythema or edema on either intact or abraded skin across all observation periods, resulting in a Primary Irritation Index (PII) of 0. This outcome definitively classifies sublancin as a “non-irritant” substance according to OECD TG 404 criteria. Crucially, the concurrent positive control (e.g., DNCB) successfully induced expected irritant responses, validating the sensitivity and proper conduct of the assay system. Similarly, in the GPMT, the complete absence of allergic reactions in sublancin-treated guinea pigs (0% sensitization rate) stands in sharp contrast to the robust sensitization (100%) induced by the positive control, 2,4-dinitrochlorobenzene (DNCB). This clear dichotomy not only confirms the assay’s capability to identify sensitizers but also provides high confidence in the negative result for sublancin. It is noteworthy that while DNCB provoked severe local skin reactions, it did not significantly affect systemic parameters like body weight in this model, underscoring the localized nature of contact hypersensitivity and reinforcing that the observed safety of sublancin is not due to a generalized insensitivity of the test animals [18,19].

The favorable safety outcome can be rationalized through an analysis of sublancin’s physicochemical and structural properties, which likely minimize its interaction with skin biology pathways, leading to adverse effects. As a glycosylated peptide with a molecular weight of approximately 3.8 kDa [15,20], sublancin exceeds the typical size threshold (<500 Da) considered optimal for passive transdermal penetration [21]. Its complex, amphiphilic cyclic structure, stabilized by disulfide bonds, may further impose steric and diffusional barriers to permeation through the stratum corneum [22]. This inherently low potential for dermal absorption is a critical factor. For sensitization to occur, a compound must penetrate the skin, be haptenized or recognized as an antigen, and be presented by Langerhans cells to T-lymphocytes [23,24]. The limited bioavailability of sublancin in the skin significantly reduces its opportunity to engage this immunological cascade. Similarly, irritation often involves direct cytotoxic effects or pro-inflammatory interactions with epidermal cells; minimal penetration likely circumvents these processes. This perspective is conceptually supported by preliminary pharmacokinetic data (unpublished), which suggest low systemic bioavailability for sublancin via other routes and hint at a general pattern of low membrane permeability that may extend to the skin. These data are cited for mechanistic plausibility only and were not used for quantitative interpretation. Therefore, the observed safety is not coincidental but appears rooted in a predictable combination of its substantial molecular size and complex conformation.

From a regulatory standpoint, the results provide the necessary data to classify sublancin as a “non-irritant” and “non-sensitizer” under the OECD guidelines for skin irritation and sensitization testing and the corresponding criteria of the Globally Harmonized System of Classification and Labelling [25]. This classification is a pivotal component of the non-clinical safety dossier required for the registration of any new veterinary topical product.

It is essential to contextualize these dermal findings within the broader toxicological assessment of sublancin. The favorable local tolerance observed herein aligns coherently with and is reinforced by other toxicological studies. Notably, independent research has demonstrated an absence of systemic toxicity even at very high oral doses in rodent models [12]. Furthermore, evaluations for genotoxic potential (e.g., Ames test, micronucleus assay) have yielded negative results. This consistency across diverse endpoints, from local tolerance to systemic toxicity and genetic safety, creates a convergent and robust weight-of-evidence for the compound’s overall low toxicological risk. The present study specifically addresses the relevant exposure route for intended applications, thereby filling a key data gap and complementing the existing safety database to sketch a more complete and reassuring non-clinical safety profile.

The demonstrated safety, particularly its maintenance on abraded skin, carries significant practical value for intended veterinary uses. In dairy farming, maintaining teat skin integrity is paramount for preventing mastitis. While traditional biocides like iodophors are effective, some can cause skin dryness or irritation with prolonged use, potentially compromising the physical barrier [26]. Sublancin’s non-irritating property suggests its potential as an alternative that could provide effective antimicrobial activity while better preserving skin health, which is crucial for animal welfare and milk quality [27,28]. Similarly, in managing wounds in livestock (e.g., from hoof lesions or procedures), topical agents must avoid causing additional pain or contact dermatitis [26]. The safety demonstrated on abraded skin in this study supports the potential tolerability of sublancin in such wound care scenarios.

This application’s potential is highly relevant within the global strategy of seeking antibiotic alternatives to combat resistance. Antimicrobial peptides (AMPs), as natural defense molecules, often possess mechanisms of action that make resistance development less likely compared to conventional antibiotics [29,30]. The progression of other AMPs, like the PL-5 spray for human wound infections [31], illustrates the translational pathway. Sublancin, with its documented in vitro antimicrobial efficacy against relevant pathogens [8,9,10,11,32,33], combined with the in vivo topical safety established here, presents a compelling candidate for development. However, it is critical to distinguish between demonstrated safety in a controlled test and proven efficacy in complex field conditions. The current data robustly support the former and justify further investment into the latter.

This work provides foundational safety data for the topical application of sublancin, yet certain limitations inherent to its design must be acknowledged to contextualize the findings and direct future veterinary product development. A primary consideration is the use of standard rodent and lagomorph models. While essential for regulatory safety screening, interspecies differences in skin thickness, immune response, and metabolism may affect the precise extrapolation of these results to all target livestock species, such as cattle or swine. Furthermore, the study evaluated sublancin as a purified entity. In a practical veterinary setting, its compatibility with necessary formulation excipients (e.g., emulsifiers, preservatives), its stability in final products, and its interaction with complex environmental factors on farms remain to be characterized.

Therefore, subsequent research should logically progress through a translational pipeline specific to veterinary medicine. The immediate step involves confirmatory safety and efficacy testing in relevant target species under conditions mimicking intended use. Concurrently, applied research must focus on developing stable, user-friendly formulations (e.g., long-acting teat dips, adhesive wound sprays) and rigorously assessing their local tolerance and physical stability [34]. Finally, to complete the safety dossier and define practical use parameters, systematic local pharmacokinetic and residue studies in target species are indispensable. Quantifying dermal absorption and depletion kinetics is critical for establishing scientifically justified withdrawal periods and maximum residue limits, which are fundamental for regulatory approval and consumer safety [35].

## 5. Conclusions

This study confirmed through guinea pig maximization tests and rabbit skin irritation tests that the antimicrobial peptide Sublancin does not have skin sensitization or irritation under the test conditions. Specifically, no skin lesions (erythema or edema) were observed in any of the 20 guinea pigs (sensitization rate 0%), and all 12 rabbits showed a total irritation score of 0 for both intact and abraded skin. According to relevant evaluation criteria, it can be determined that the skin sensitization and irritation test results of Sublancin are negative, which provides core experimental support for its local application safety. These findings support the progression of sublancin to safety and efficacy evaluations in target animal species, marking a key translational step toward its potential development as a topical veterinary agent.

## Figures and Tables

**Table 1:** Scoring criteria for skin allergic reactions.

Skin Reaction	Intensity	Score
Erythema	No erythema	0
	Mild erythema (barely visible)	1
	Moderate erythema (clearly visible)	2
	Severe erythema	3
	Purple-red erythema to slight eschar formation	4
Edema	No edema	0
	Mild edema (slight raising)	1
	Moderate edema (distinct raising)	2
	Edematous erythema	3

**Table 2:** Classification of skin sensitization intensity.

Skin Reaction (%)	Grade	Intensity
0–8	Ⅰ	Weak
9–28	Ⅱ	Mild
29–64	Ⅲ	Moderate
65–80	Ⅳ	Strong
81–100	Ⅴ	Extreme

Note: A sensitization rate of 0% is interpreted as no skin allergic reaction observed. The sensitization rate was calculated as follows: (Number of animals exhibiting erythema or edema/Total number of animals in the group) × 100%.

**Table 3:** Scoring Criteria for Skin Irritation Reactions.

Irritation Response	Description of Intensity	Score
Erythema (A)	No erythema	0
	Mild erythema (barely visible)	1
	Moderate erythema (clearly visible)	2
	Severe erythema	3
	Erythema with eschar formation	4
Edema (B)	No edema	0
	Mild edema (barely visible)	1
	Moderate edema (distinct raising with clear outline)	2
	Severe edema (raised >1 mm)	3
	Severe edema (raised >1 mm and extensive)	4
Highest total score (A + B)		8

**Table 4:** Evaluation criteria for skin irritation intensity.

Mean Score (A + B)	Evaluation
0–0.5	Non-irritant
0.6–3.0	Mildly irritant
3.1–6.0	Moderately irritant
6.1–8.0	Severely irritant

Note: The Mean Score (A + B) is the average total irritation score (Erythema + Edema, from Table 3) per group across all animals and observation times. Classification thresholds are conventionally derived from the OECD Guideline 404 skin reaction scoring system.

**Table 5:** Skin allergy reaction scores and sensitization rates in guinea pigs.

Time Point (h)	Group	N	Erythema Score (No. of Animals)					Edema Score (No. of Animals)				Sensitization Rate (%)
			0	1	2	3	4	0	1	2	3	
24	Positive Control	10	0	0	4	6	0	0	0	3	7	100
	Negative Control	10	10	0	0	0	0	10	0	0	0	0
	Sublancin-Treated	20	20	0	0	0	0	20	0	0	0	0
48	Positive Control	10	0	0	7	3	0	0	0	4	6	100
	Negative Control	10	10	0	0	0	0	10	0	0	0	0
	Sublancin-Treated	20	20	0	0	0	0	20	0	0	0	0
72	Positive Control	10	0	0	7	2	1	0	0	4	6	100
	Negative Control	10	10	0	0	0	0	10	0	0	0	0
	Sublancin-Treated	20	20	0	0	0	0	20	0	0	0	0

**Table 6:** Skin irritation response scores in rabbits following single application on intact skin.

Time Point (h)	Rabbit ID	Erythema (A)		Edema (B)		Total Score (A + B)	
		Negative Control	Sublancin-Treated	Negative Control	Sublancin-Treated	Negative Control	Sublancin-Treated
1	1	0	0	0	0	0	0
	2	0	0	0	0	0	0
	3	0	0	0	0	0	0
24	1	0	0	0	0	0	0
	2	0	0	0	0	0	0
	3	0	0	0	0	0	0
48	1	0	0	0	0	0	0
	2	0	0	0	0	0	0
	3	0	0	0	0	0	0
72	1	0	0	0	0	0	0
	2	0	0	0	0	0	0
	3	0	0	0	0	0	0

Note: The scores for both the application and control sites of all rabbits were 0 at all time points. According to the criteria in Table 4, sublancin is determined to be non-irritating to the intact skin of rabbits. The same applies to the tables below.

**Table 7:** Skin irritation response scores in rabbits following single application on abraded skin.

Time Point (h)	Rabbit ID	Erythema (A)		Edema (B)		Total Score (A + B)	
		Negative Control	Sublancin-Treated	Negative Control	Sublancin-Treated	Negative Control	Sublancin-Treated
1	1	0	0	0	0	0	0
	2	0	0	0	0	0	0
	3	0	0	0	0	0	0
24	1	0	0	0	0	0	0
	2	0	0	0	0	0	0
	3	0	0	0	0	0	0
48	1	0	0	0	0	0	0
	2	0	0	0	0	0	0
	3	0	0	0	0	0	0
72	1	0	0	0	0	0	0
	2	0	0	0	0	0	0
	3	0	0	0	0	0	0

**Table 8:** Skin irritation response scores in rabbits following repeated application on intact skin.

Time Point (h)	Rabbit ID	Erythema (A)		Edema (B)		Total Score (A + B)	
		Negative Control	Sublancin-Treated	Negative Control	Sublancin-Treated	Negative Control	Sublancin-Treated
1	1	0	0	0	0	0	0
	2	0	0	0	0	0	0
	3	0	0	0	0	0	0
24	1	0	0	0	0	0	0
	2	0	0	0	0	0	0
	3	0	0	0	0	0	0
48	1	0	0	0	0	0	0
	2	0	0	0	0	0	0
	3	0	0	0	0	0	0
72	1	0	0	0	0	0	0
	2	0	0	0	0	0	0
	3	0	0	0	0	0	0

**Table 9:** Skin irritation response scores in rabbits following repeated application on abraded skin.

Time Point (h)	Rabbit ID	Erythema (A)		Edema (B)		Total Score (A + B)	
		Negative Control	Sublancin-Treated	Negative Control	Sublancin-Treated	Negative Control	Sublancin-Treated
1	1	0	0	0	0	0	0
	2	0	0	0	0	0	0
	3	0	0	0	0	0	0
24	1	0	0	0	0	0	0
	2	0	0	0	0	0	0
	3	0	0	0	0	0	0
48	1	0	0	0	0	0	0
	2	0	0	0	0	0	0
	3	0	0	0	0	0	0
72	1	0	0	0	0	0	0
	2	0	0	0	0	0	0
	3	0	0	0	0	0	0

## Data Availability

The data presented in this study are available on request from the corresponding author.

## Supplementary Materials

The following supporting information can be downloaded at: https://www.mdpi.com/article/10.3390/toxics14010069/s1, Figure S1: Schematic representation of sublancin’s structure; Table S1: The weight gain of guinea pigs in different groups over 4 weeks.

## Abbreviations

The following abbreviations are used in this manuscript:

GPMT	Guinea Pig Maximization Test
AMPs	antimicrobial peptides
NOEL	No-Observed-Effect Level
NMR	nuclear magnetic resonance
DNCB	2,4-dinitrochlorobenzene
ANOVA	one-way analysis of variance
PII	primary irritation index
