# Supplementary material for: Antimicrobial Peptide Sublancin Skin Sensitization and Irritation Assessment in Guinea Pigs and Rabbits
Source: Toxics. 2026 Jan 12;14(1):69. doi: 10.3390/toxics14010069 (PMC12846217; doi:10.3390/toxics14010069)
Supplement: Supplementary file 1 [file toxics-14-00069-s001.zip › toxics-4058128-Supplementary.pdf]

toxics

Supplementary Data

Antimicrobial Peptide Sublancin Skin Sensitization and Irritation Assessment in Guinea Pigs and Rabbits

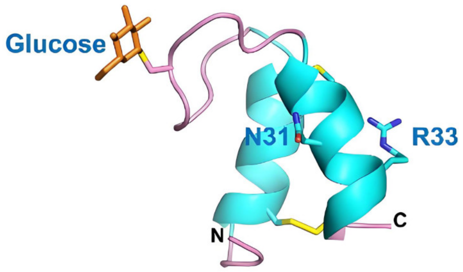

Figure S1. Schematic representation of sublancin's structure.

Table S1. The weight gain of guinea pigs in different groups over 4 weeks.

Groups		Negative CON		Positive CON		Sublancin	
Experimental rats		♀ 5	♂ 5	♀ 5	♂ 5	♀ 10	♂ 10
Average Body weight of rats during each experimental period (g/day/rat)	1-7 day	2.68±1.15	4.00±1.52	3.14±0.75	4.18±1.36	3.01±1.32	4.44±1.63
	8-14 day	2.96±1.06	3.34±0.86	3.66±1.21	4.30±0.85	3.08±1.43	4.01±1.61
	15-21 day	3.16±0.59	3.44±0.98	3.38±1.51	3.46±1.37	2.99±1.55	3.54±1.27
	22-28 day	3.18±1.23	3.26±1.12	3.74±1.48	4.00±1.93	3.13±1.08	3.21±1.42

Note: The data were compared for statistical significance with the same-sex control group. In the same row, values with different small letter superscripts mean significant difference ($P<0.05$), while with the same or no letter superscripts mean no significant difference ($P>0.05$). The same as below. Negative CON group, basal diet; Positive group, basal diet + 2,4-dinitrochlorobenzene; Sublancin group, basal diet + Sublancin.
